# Cellulose Mediated Transferrin Nanocages for Enumeration of Circulating Tumor Cells for Head and Neck Cancer

**DOI:** 10.1038/s41598-020-66625-2

**Published:** 2020-06-19

**Authors:** Raj Shankar Hazra, Narendra Kale, Gourishankar Aland, Burhanuddin Qayyumi, Dipankar Mitra, Long Jiang, Dilpreet Bajwa, Jayant Khandare, Pankaj Chaturvedi, Mohiuddin Quadir

**Affiliations:** 1 Department of Mechanical Engineering, Materials and Nanotechnology Program, North Dakota State University, Fargo, 58108 ND USA; 2 Department of Coatings and Polymeric Materials, North Dakota State University, Fargo, 58108 ND USA; 3 Maharashtra Institute of Technology-WPU, School of Pharmacy, Pune, India; 4 Actorius Innovations and Research (AIR) Pvt. Ltd., Pune, India; 5 Department of Electrical and Computer Engineering, North Dakota State University, Fargo, 58108 ND USA; 6 Department of Medical Oncology, Tata Memorial Hospital, Mumbai, 400012 Maharashtra India

**Keywords:** Biosensors, Cancer screening

## Abstract

Herein we report a hierarchically organized, water-dispersible ‘nanocage’ composed of cellulose nanocrystals (CNCs), which are magnetically powered by iron oxide (Fe_3_O_4_) nanoparticles (NPs) to capture circulating tumor cells (CTCs) in blood for head and neck cancer (HNC) patients. Capturing CTCs from peripheral blood is extremely challenging due to their low abundance and its account is clinically validated in progression-free survival of patients with HNC. Engaging multiple hydroxyl groups along the molecular backbone of CNC, we co-ordinated Fe_3_O_4_ NPs onto CNC scaffold, which was further modified by conjugation with a protein - transferrin (Tf) for targeted capture of CTCs. Owing to the presence of Fe_3_O_4_ nanoparticles, these nanocages were magnetic in nature, and CTCs could be captured under the influence of a magnetic field. Tf-CNC-based nanocages were evaluated using HNC patients’ blood sample and compared for the CTC capturing efficiency with clinically relevant Oncoviu platform. Conclusively, we observed that CNC-derived nanocages efficiently isolated CTCs from patient’s blood at 85% of cell capture efficiency to that of the standard platform. Capture efficiency was found to vary with the concentration of Tf and Fe_3_O_4_ nanoparticles immobilized onto the CNC scaffold. We envision that, Tf-CNC platform has immense connotation in ‘liquid biopsy’ for isolation and enumeration of CTCs for early detection of metastasis in cancer.

## Introduction

Early detection is the key to efficacious therapeutic outcome for a number of cancers including those of epithelial origins, such as lung, colorectal and breast^1^. Occurrence of circulating tumor cells (CTCs) in peripheral blood serves as an indicator for dismal clinical prognosis in chemo-naive patients in breast cancer^2^. Thus, CTCs enable the monitoring of disease progression and is a biomarker for predicting short survival percentage in metastatic cancers, including breast, prostate and colorectal cancers. Facile, low-cost and relatively rapid detection of CTCs in patients’ blood is an urgent and unmet need for combating cancer. Unlike other systemically driven cancers like breast, lung and colorectal, HNC is considered as a locoregional cancer. Thus, the overall survival (OS) and progression free survival (PFS) in HNC patients is comparatively higher than other systemically spreading epithelial cancers. Even with the intensive concomitant chemo-radiotherapy, the locoregional recurrence or systemic failure in HNC patients is up to 50% in all stages combined. The median survival period of obstinate recurrent HNC is very short and is of 12 months, and remains unchanged for past few decades^3–6^. More studies in this direction are highly imperative, especially with the relevance of CTCs and their role in different tumor site pathogenesis.

CTCs are known to follow established ‘seed and soil’ theory of Paget in their dissemination from primary tumor site, and migration through systemic circulation^7,8^. Eventually, CTCs are hypothesized to colonize the distant tissues leading to the formation of a new tumor microenvironment^9^. Challenges associated with identification and detection of micrometastasis is mostly due to extremely low abundance of CTCs, as low as <0.1% in peripheral blood^10^. Successful detection of CTCs at such low abundance not only depends on the capacity of a diagnostic device to capture the CTCs, but also to detect and distinguish the signals originating from interactions of the device with extremely heterogeneous cellular background^11^. Currently, microfluidic chips are used to isolate CTCs, showing 65–95% of capture efficiency^12–14^. Such microfluidic platform consists of microposts array, which are grafted with anti-EpCAM antibodies. Ligands are functionalized within the microfluidic channels to isolate CTCs from the blood stream. For example, Chen *et al*. reported a hybrid magnetically deformable CTC microfluidic filter assay chip, which can capture CTCs with 85% capture efficiency from HCT116 cell line^15^. One of the major limitations of this technique is the reduction of capture efficiency with increment of blood flow as the fluid flow rate critically direct the duration of cell–micropost contact^12,16,17^. Shear force in capillary flow channel platform must be low to confirm the maximum attachment of CTCs, which in return takes much longer time to isolate. Furthermore, the efficiency of such device to isolate CTCs from large volume of blood is limited because full sample volume does not come in contact with active channel walls. Currently studied negative immune-magnetic enrichment technique does not depend on the biomarker expression of CTCs^18^. The main limitation of this process is the requirement of large amount of antibodies to isolate CTC, which renders the process expensive with low purity and lower specificity^19,20^. EasySep Human CTC Enrichment kit from Stem cell Technologies are also used to capture CTCs which showed 79% capture efficiency using CD45 transmembrane protein^21^. Membrane microfilter-based technique has also been reported to capture CTCs, where ligands are functionalized on the polymer based membrane filter to capture CTCs from blood flowing through the small size pore in the polymer matrix, and show 80% capture efficiency^22^. As the pore sizes of these membranes are very small, micro-filter gets clogged in higher flow rate, causing low capture efficiency^23–25^. Density based separation technique have also been used to capture CTCs, where ligands are designed and attached to the surface of microbead to isolate CTC by targeted sedimentation of microbead-CTC complex^26^. Yoo *et al*. demonstrated that CTCs can be isolated with the help of transparent silica microbeads^26^. In this process, precise detection of captured CTCs is very complicated as free microbeads are also mixed alongside with CTC bound microbeads. Apart from that, microbeads also interfere with the fluorescence signal of CTC detection due to significant amount of light scattering^26,27^. Immunochemistry-based techniques, such as CellSearch and MagSweeper can identify and enumerate CTCs from the whole blood^28,29^. MagSweeper is used to isolate CTCs from blood using EPCAM biomarker, which can separate high-purity cells but with maximum 62% capture efficiency^30,31^. Recently, Strep-tag technique is studied to isolate CTCs with 79% of capture efficiency using EpCAM, HER2, EGFR biomarker and magnetic beads, which showed CTC enrichment under external magnetic field with very fast isolation^32^. This process relies on an expensive EpCAM biomarker, which leads expensive detection and limits its wide-spread use^33–35^. Wen *et al*. proposed similar magnetic nanospherical modules, prepared by an emulsifier-free polymerization method coupled with layer-by-layer assembly to capture rare tumor cells^36^. Zhou *et al*. demonstrated Quantum dots (QDs) decorated with PEG to form immuno-magnetic nanospheres to capture CTC using EpCAM antibody^37^. In a similar study, Xie *et al*. designed a composite material prepared by encapsulating a magnetic iron oxide core in MIL-100 shell that is conjugated with anti-EpCAM to capture CTCs from blood^38^. CTC capturing using EpCAM antibody has limitations, particularly with the population of circulating cells that does not present EpCAM protein, or those which went through epithelial-to-mesenchymal transition^39–41^. For these reasons, the antibody-coated magnetic platform using EpCAM may be at dismal to detect CTCs^42^, and only 70% tumor cells out of 134 types could be detected and a major segment of invasive tumor cells remains undetected by EpCAM^43,44^. In addition to antibody-based CTC enumeration, there is a need to investigate capability of other molecular markers to isolate CTCs. For example, CTC’s EMT transition leading to overexpression of extracellular matrix vimentin and fibronectin could also be targeted by other ligands. Capturing CTCs with a materials platform using transferrin (Tf) as a capture ligand have been studied widely due to higher concentration of Tf receptor in tumor cells compared to healthy cells^45–47^. As a transmembrane glycoprotein, Tf receptors are highly expressed in cancer cells i.e., about 100-fold higher than healthy cells^48–50^. Major role of Tf receptor is to transport iron in living cells and in case of cancer cells, this receptor is highly expressed due to the elevated requirement of iron to support cellular survival^48–50^. Transferrin (Tf) has demonstrated specific and efficient identification of Tf-receptors in cancer cells irrespective of EMT status^40,48,51–53^. Recent work has demonstrated a nanogel system, functionalized with Tf conjugated polyethylene glycols (PEGs) linker to capture CTCs from blood^39^. Zhang *et al*. have used Tf conjugated silica-based nanoparticles to identify CTCs, using fluorescence properties^48^. Thus, our focus remains on Tf-mediated CTC capturing with more efficient nanosystems^11,54^. Furthermore, we reported earlier a multivalent, magneto-dendritic, nanoparticle (NP) systems conjugated with Tf that demonstrated superior CTC capture efficiency from peripheral blood cells. It was observed that in addition to stability in blood, size and shape of the magnetic nanoparticles, as well as their mechanical properties, such as elasticity and geometry, and spatial arrangement of cell-specific ligands on particle surface critically control their capacity to interact with CTCs^39,55–59^. Most of the CTC capture platforms reported thus far by us and others are either spherical or branched architectures, constructed as nanoparticles with an aspect ratio nearly 1.0 that tend to limit CTC capturing specificity and efficiency due to finite number of ligands that can be accommodated onto such particle surface. We hypothesize that, a high aspect ratio; water-dispersible, nanometer scale polymeric scaffold decorated with Tf can provide augmented multivalency and contact points conducive for efficient ligand-receptor interactions, thereby improving the affinity of the diagnostic constructs towards CTCs. To prove this hypothesis we selected to use high aspect ratio nanostructures such as cellulose nanocrystals (CNCs) possessing multivalent hydroxyl functional groups, which were chemically modified to co-ordinate magnetic iron oxide (Fe_3_O_4_) NPs. Cellulose-derived materials are the most abundant biopolymer obtained from renewable resources and demonstrate a repertoire of attractive physico-chemical and materials properties, including but not limited to, biocompatibility, biodegradability and environmental sustainability. The advantage of CNC is its high aspect ratio, large specific surface area and superior quantity of reactive surface functional groups^60^. The reason behind the usage of cellulose nanocrystal are due to the ease of functionalization, as it has large number of hydroxyl group present on its surface; ease of purification as compared to other nanosystems and better dispersibility along with high loading capacity of biological components, and non-toxicity^60^. Facile and orthogonal chemical conjugation of the reactive and regularly arranged hydroxyl groups along CNC structures with functional substrates enabled the formation of a wide variety of CNC-derivatives with unique solution properties such as ease of processing, tunable flexibility, superior mechanical properties, electrical conductivity, and affinity towards other biomolecules in multivalent fashion^61,62^. Regularly spaced reactive hydroxyl groups along CNCs provide excellent steric selectivity that can be utilized for preparing optical and enzymatic biosensors, and the carbohydrate nature of the cellulose nanocrystal, leads to non-sticky surface, which in turn helps to inhibit adsorption of non-targeted analyte^60^. As CNC has large number of hydroxyl groups on its surface, which can be modified to change its hydrophilicity, it is easy to form stabilization matrix with CNC for conjugating metallic nanoparticles that helps to bind with specific protein and biomarkers for targeted biomedical applications^63–65^. For example, excellent solid fluorescent materials have been developed through covalent attachment of luminogens onto cellulose backbone^66^. Recently, Schyrr *et al*. have demonstrated CNC- and PVA-based platform for use in fluorescence-based biomedical applications^67^ and Incani *et al*. have also developed glucose sensors by immobilization of glucose oxidase (GOx) on the cellulose nanocrystal PEI/AuNPs composite^60^. Similarly, Saeed *et al*. proposed a technique with gold nanoparticle decorated functionalized cellulose nanocrystal and immobilized GOx enzyme to sense salivary glucose^68^. Efforts were made for developing point of care biosensors by using conjugation of peptides and protein to CNCs^64,65^. In this project, native hydroxyl functional groups on CNCs were first converted to amines, which were then covalently modified to thiol to coordinate with iron oxide nanoparticles and the resulting substrate was partially conjugated with Tf. Superparamagnetic Fe_3_O_4_ NPs were designed through hydrothermal route and endorsed into magnetic isolation of Tf-engaging cells. Hierarchically organized networks similar to ‘nanocages’ were obtained through subsequent crosslinking of modified CNCs with these Fe_3_O_4_ nanoparticles, where magnetic NPs were homogeneously distributed throughout the network. In our work we showed that, the CNC-derived materials provided a matrix with hydrophilic properties, aqueous dispersibility, colloidal stability and cellular compatibility and CTC capture efficiency. This report describes synthetic methodology, characterization, and comparative clinical performance evaluation of these magnetic NP stabilized, high-aspect ratio cellulose network for capturing CTCs in HNC patients. Furthermore, the CTC enumeration efficiency using CNC nanocage systems was compared with clinically developed Oncoviu platform^69^.

## Results

### Design of CNC-derived CTC-capture platform

**Figure 1:**
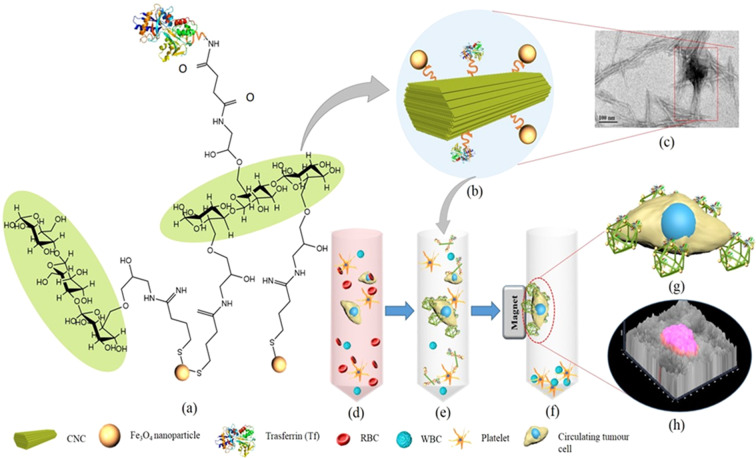
CNC nanocage depicting the capturing of CTCs mediated by Tf-conjugation and coordination of Fe_3_O_4_ NPs. (**a**) Chemical structure of Tf conjugated and Fe_3_O_4_ immobilized CNCs, **(b**) schematic representation of Tf conjugated and Fe_3_O_4_ immobilized CNC, (**c**) TEM image representing Fe_3_O_4_ co-ordinated CNC nanocages. Schematic illustration of the process (**d**) blood sample containing a mixture of blood cells (RBC, WBC and platelets) and CTCs collected from cancer patient, (**e**) collected blood samples treated with RBC lysis buffer to remove RBC and surface receptors thereof, and centrifuged. Resultant pellet re-suspended in PBS buffer and exposed to synthesized iron oxide and Tf conjugated CNC. (**f**) In the presence of magnetic field Tf-receptor enriched CTCs were captured by Tf-CNC nanocage and were attracted towards the magnet, while other cellular components settle down. (**g**) Illustration of CTC captured by CNC nanocage, (**h**) 2.5D merged image of cancer cells captured by CNC nanocage.

We designed and synthesized a cross-linked, high aspect ratio CNC platform decorated with Tf and stabilized by magnetic NPs. CNCs were obtained by acid hydrolysis of cellulose by dissolving the amorphous region of the biopolymer. We used CNC-based materials due to the presence of large number of hydroxyl groups, which are amenable to functionalization and ease of conversion into a self-assembled, hierarchical network structure. We also envisioned that, intrinsic micromechanical and chemical properties, such as geometry, rigidity and hydrophilicity provided by CNC could result in superior cell entrapment and non-lethal capture properties of the diagnostic scaffold. The schematic mechanism of CTC capturing technique by magnetized and ligand-decorated CNC materials is presented in Fig. 1. To obtain this multi-functional structure, we first modified CNCs with appropriate reactive groups, which were then immobilized with Fe_3_O_4_ NPs and Tf at different stoichiometric ratio.

### Functionalization of CNCs

**Figure 2:**
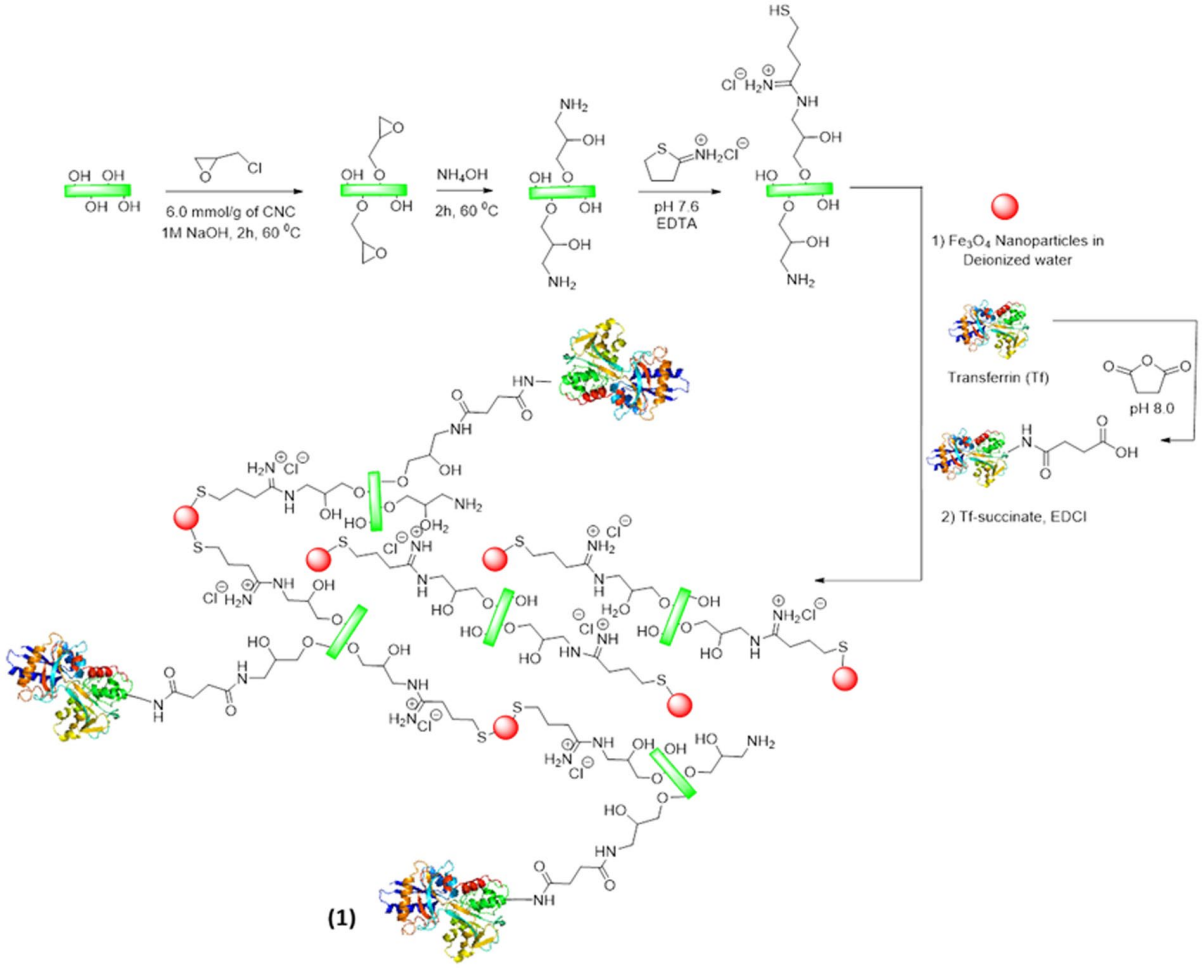
Synthetic scheme of amine and thiol functionalization of CNCs. Fe_3_O_4_ was conjugated to CNC-amines through thiol linkages generated on primary amines of CNC. Remaining amine groups of CNCs were connected to Tf through succinyl moiety.

Multi-step functionalization of CNCs has been carried out to stabilize Fe_3_O_4_ and Tf onto the CNC scaffold to yield the cellulose nanocage platform (as shown in structure **1** in Fig. 2). One of the major challenges of modifying CNCs is their extensive hydrogen bonding capacity. Only a selected set of organic solvents, or buffered aqueous solutions are generally used to carry out CNC modifications as evident from literature^70^. Thus, we have adopted an epichlorohydrin-mediated functionalization of CNC hydroxyl groups for their conversion to amines following the protocol reported by Dong *et al*.^71^. This is to mention that commercially available CNCs were modified before the chemical reaction by repetitive washing procedure with water to remove acidic impurities. We have targeted three different functionalization levels (in mmols of hydroxyl groups per g of cellulose basis). Following alkaline hydrolysis and extensive dialysis, amine functionalized CNC materials were obtained in quantitative yield. We have treated CNC samples with different amount of epichlorohydrin (12, 24 and 48 mmol/g CNC) to prepare CNC-NH_2_ at different functionalization levels (coded as F-1, F-2 and F-3 respectively).

Since the generation of CNC-NH_2_ from pristine CNCs is the key to the formation of nanocages, we have extensively characterized the amine functionalization chemistry of CNCs using UV-Vis, infrared (IR), X-ray photoelectron spectroscopy (XPS) and by elemental analysis. The generation of amine groups onto CNC surface was first validated by reacting purified CNC-NH_2_ with fluorescein isothiocyanate (FITC). Isothiocyanate group of FITC reacts exclusively with amines, and such reaction of FITC served as quantitative approach to identify the amine loading on CNCs. We observed that, FITC conjugation resulted in the formation of a yellow homogenous solution at pH 6 after dialysis for 5 days, indicating the presence of only covalently connected FITCs to amines generated on CNCs. The UV-Vis spectra for FITC functionalized CNC-NH_2_ at different functionalization level (F1-F3) provided significant molecular characterization data (Supplementary Figure 1a). The spectra were recorded from 200 nm to 600 nm spectral range where the absorption peak was found at 493 nm owing to the presence of dianionic, while peak at 455 nm and 473 nm were observed due to anionic form of FITC^71^. Both dianionic and anionic peak confirms the presence of FITC onto CNC surface. It is also evident that the intensity of absorption peak is increased by ~1.9 folds as the level of amine functionalization was increased from formulation F-1 to F-3. Since, FITC has high affinity towards amine functional groups, gradual increments of intensity suggested the increment in the level of amine functionalization onto CNC surface and success of such chemical conversion.

We also confirmed the presence of amine functionalization onto CNC surface by infrared (IR) spectroscopy, which clearly depicts a broad spectral band at 3000–3550 cm^−1^ that arises due to overlap of O-H and N-H stretching (Supplementary Figure 1b) corresponding to the formation of CNC-NH_2_ derivative. Another relevant distinct spectral band is found at 1631 cm^−1^, which arises due to N-H bending^71,72^. The absorption at 1631 cm^−1^ is prominently increased by ~9 folds from low (F-1) to high level of functionalization (F-3) of CNCs to CNC-NH_2_, suggesting increment of the number of amine groups as the level of functionalization was increased. X-ray photoelectron spectroscopy (XPS) has also been conducted to confirm the presence of nitrogen in functionalized CNC-NH_2_ samples (Supplementary Figure 1c). The comparative spectra between the pristine CNC and CNC-NH_2_ shows that the peak for N1s arises due to the presence of O=C-N group after functionalization^73,74^. The plot with high magnification scans of N1s peak is shown in the inset of Supplementary Figure 1c, where the peak position is found to be at 399 eV, extracted by Lorentzian fitting. The elemental analysis by combustion using automatic analyser has also conducted on the modified CNC samples. Elemental analysis data demonstrated a clear trend of the CNC modification as a function of epichlorohydrin/ammonium hydroxide treatment, as indicated by the difference of N/C ratio for different functionalization levels (Supplementary Figure 1d). This result shows that N/C ratio is increased by 67% from low (F-1) to medium (F-2) functionalization and by 33% from medium (F-2) to high (F-3) functionalization.

### Immobilization of Fe_3_O_4_ NPs on CNC-NH_2_

Once we confirmed the successful functionalization of CNCs with amines, we coordinated Fe_3_O_4_ NPs to CNC scaffold. We adopted the well-established hydrothermal method as reported earlier to prepare Fe_3_O_4_ NPs^75^. To achieve iron oxide nanoparticles of homogenous polydispersity, both ferrous and ferric chloride salts were mixed following incubation with NH_4_OH dropwise. While the pH of this solution was maintained at 10.0, the precipitated nanoparticles were heated up to 80 °C and purified by centrifugation (3×). The process resulted in homogenous dispersion of iron oxide nanoparticles, which were found to be within the size range of 80–140 nm as determined by transmission electron microscopy (TEM) and dynamic light scattering (DLS) as shown in Supplementary Figure 2a (crystal plane of encaged Fe_3_O_4_ is shown in Supplementary Figure 2b). For attachment of iron oxide nanoparticles with CNC-NH_2_, we have partially functionalized amine groups located on CNC-surfaces to thiol by treating the CNC-NH_2_ samples with 2-iminothiolane at increasing molar ratio with respect to the amine group. Popularly known as Traut’s reagent, iminothiolane quantitatively attaches to amino groups to generate a sulfhydryl (−SH) end group. Traut’s reaction has been extensively studied in bio-conjugation, and was found to be affected by pH and time of reaction^76,77^. To minimize disulfide bond formation, and maximize the yield of thiol-modified product, we have optimized the reaction conditions at pH of 7.6, and spiked with EDTA to complex divalent metal ions (for inhibiting disulfide bond formation), and limited the reaction time to 45 minutes. Due to high binding constants between thiol group and Fe_3_O_4_, we simply incubated Fe_3_O_4_ NPs in DI water (at a concentration of 5.3 mg/mL) with thiol modified CNC-NH_2_ suspension under bath sonication over 20 minutes to generate Fe_3_O_4_ NP stabilized CNC-nanocage network. Different synthetic formulations of nanocage constructs fabricated in the above-mentioned process, which are presented in Supplementary Table-1. As expected, we observed increasing amount of Fe_3_O_4_ can be immobilized as thiol concentration is increased on a CNC scaffold at a fixed quantity of amines. In the following steps, post-functionalization of Fe_3_O_4_-CNC amines with succinic anhydride enabled conjugation of Tf, which enabled formation of Tf immobilization onto CNC nanocages. Since remaining primary amines of CNC were used to immobilize Tf, post Fe_3_O_4_ nanoparticle conjugation, the amount of Tf immobilized on the final CNC product do not show a linear dependence on initial amine functionalization level of CNCs (Supplementary Table-1). Quantity of Fe_3_O_4_ were measured by UV-Vis analysis with the help of a calibration curve (Supplementary Figure 3), while amount of Tf were enumerated with the help of modified Bradford assay (Supplementary Figure 4).

**Figure 3:**
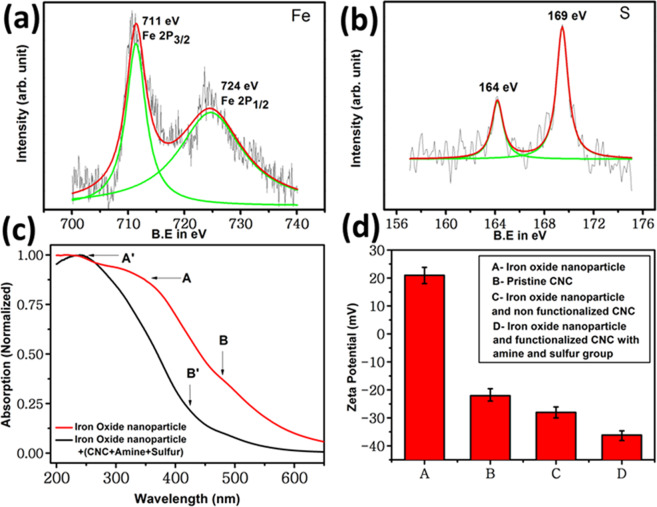
Iron oxide nanoparticle bonding with modified CNC samples. (**a,b**) XPS spectra of modified CNC sample after iron oxide nanoparticle attachment showing presence of iron and sulfur bonding (thiol) respectively. Lorentzian fitting of the peaks is shown in green. (**c**) Comparative UV-Vis spectroscopy of pristine iron oxide nanoparticle and after CNC attachment. (**d**) Zeta potential of (A) pristine CNC at pH 7.0; (B) pristine Fe_3_O_4_ NPs at pH 5.0; (C) Fe_3_O_4_ NPs mixed with pristine CNC at pH 7.0; and (D) iron oxide nanoparticles attached with thiol functionalized modified CNC at pH 7.0.

To validate the formation of CNC-metal NP constructs, XPS analysis of the resulting materials has been conducted which are presented in Fig. 3a,b respectively. Two distinct relevant peaks were found (Fig. 3a) at 711 eV and 724 eV, which arises due to Fe 2P3/2 and Fe 2P1/2 respectively^78,79^. These iron peaks are introduced only after immobilization of Fe_3_O_4_ in modified CNC sample. Due to immobilization of sulfur groups before adding iron oxide in modified CNCs, two prominent peaks of S2p were also evident at 164 eV and 169 eV^80,81^ (Fig. 3b). The intensity ratio between these two peaks is 1:2 which clearly demonstrates the interaction between iron and sulfur is more dominant than disulfide interaction, indicating the formation of iron oxide entrapped CNC nanocages.

UV-Vis spectroscopy was performed to confirm the presence of iron oxide nanoparticles within amine modified CNC samples, that has been pre-modified with thiol. As shown in Fig. 3c, absorption peaks arises at A (344 nm) and B (480 nm) for pristine Fe_3_O_4_ NPs. These peaks were found to be shifted to A’ (239 nm) and B’ (425 nm) as these NPs being incorporated within CNC-NH_2_ network. This observed blue shift is most likely due to change in electronic band structure (increase in band gap) of Fe_3_O_4_ nanoparticles as they attach themselves to modified CNC through its lattice (Fig. 3c). This set of experiment indicates that thiolated primary amines are the major seat of Fe_3_O_4_ nanoparticles for their immobilization on to the CNC scaffold.

Zeta potential measurement demonstrated the aqueous colloidal stability of resulting Fe_3_O_4_ NPs immobilized onto CNCs, and indicated the importance of thiol modification of primary amines (Fig. 3d) on metal stabilization. Zeta potential of pristine Fe_3_O_4_ NPs at pH 5.0 and pristine CNC (pH 7.0) were found to be +21 mV and −22 mV respectively^81,82^. It was observed that, the highest charge reversal of Fe_3_O_4_ NPs were obtained (+21 to −36 mV value at pH 7.0, between sample A and D) when Fe_3_O_4_ NPs were connected to CNC nanocages via thiol groups compared to when CNC and Fe_3_O_4_ NPs were physical connected (through electrostatic and hydrophobic interaction, not mediated via thiol-metal linkage) as shown in Fig. 3d (please compare sample C and D). The anionic surface charge over the Fe_3_O_4_ -CNC construct was found to be essential for colloidal stability nanocages prepared through this thiolation step. This result also signifies that Fe_3_O_4_ NPs is conjugated to CNC samples through sulfur atom, as the mixture of pristine CNCs and Fe_3_O_4_ NPs was not stable, and Fe_3_O_4_ particularly, precipitated out of solution after few hours. We also observed multiple population of nanoparticles in this suspension where Fe_3_O_4_ NPs were physically mixed with CNC without thiol modification of the latter, showing instability of the colloidal suspension (discussed later in following sections).

### Self-assembly of Fe_3_O_4_ NP containing CNC nanocages and Tf conjugation

**Figure 4:**
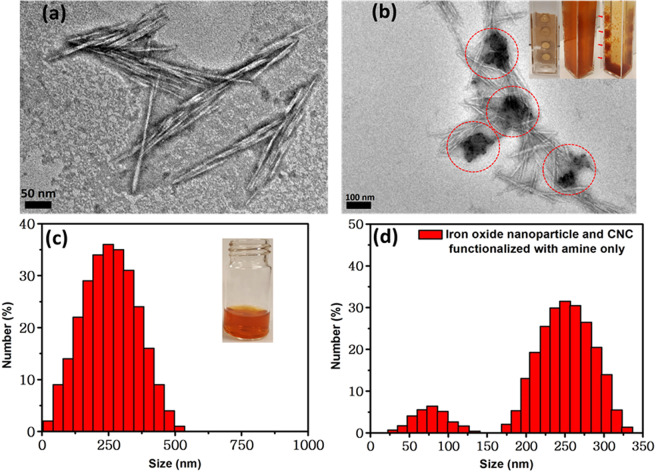
Geometry of the hierarchical nanostructures formed by Fe_3_O_4_ NPs and modified CNCs. (**a**) TEM micrograph of pristine, un-functionalized CNCs. (**b**) TEM image of conjugated with modified CNCs conjugated with Fe_3_O_4_ NPs forming ‘cage’ like hierarchical nanostructures, inset shows the paramagnetic activity of these nanoparticles in the presence of a magnet **(c**) Particle size distribution of cage like, hierarchical nanostructures formed by modified CNC immobilized with Fe_3_O_4_ NPs. In the inset, optical image of a homogenous and stable dispersion of iron oxide nanoparticles and modified CNCs. (**d**) Unmodified CNC-NH_2_ (without thiol functionalization) when mixed with Fe_3_O_4_ NPs, a colloidal dispersion was observed, which showed two distinct populations of nanoparticles with a number averaged particle size of 87 ± 6 nm and 256 ± 4 nm.

We have investigated the geometry of hierarchical nanostructures formed through conjugation of Fe_3_O_4_ NPs and modified CNCs using transmission electron microscopy (TEM) and dynamic light scattering (DLS). TEM was performed to investigate structural features of native CNCs and Fe_3_O_4_ NP modified CNCs (Fig. 4a,b). It was found that pristine CNCs were quite uniform with longer axis of about 200–230 nm, and a radius of 2–5 nm (Fig. 4a)^40^, which we expect to provide enhanced accessibility and attachment of the CNC-based nanomaterials with Fe_3_O_4_ NPs as well as to target cells. No difference in size or shape of CNCs was observed when pristine CNCs were converted to CNC-NH_2_. From Fig. 4b and Supplementary Figure 5, it is clearly seen that these high aspect ratio CNC derivatives formed very unique ‘cage’-like structures, upon conjugation with Fe_3_O_4_ NPs. We also observed that these nanocages were magnetically active (inset, Fig. 4b) as evident from the localization of the nanoconstructs across a magnetic strip placed near the solution. CNC entrapped Fe_3_O_4_ NPs resulted in the formation of a uniform stable solution with particle size of 200–300 nm, with number average particle size of 254.0 ± 6.0 nm (Fig. 4c). This homogeneous suspension suggests that Fe_3_O_4_ NPs are completely conjugated within modified CNCs and formed a stable colloidal system, stabilized by strong negative surface charge. When un-functionalized CNC-NH_2_ (without thiol modification) was mixed with Fe_3_O_4_ NPs, a colloidal dispersion was observed which showed two distinct populations of nanoparticles with a number averaged particle size of 87 ± 6 nm and 256 ± 4 nm (Fig. 4d). The two populations of nanoparticle sizes found from DLS analysis (Fig. 4d) are due to the presence of unconjugated Fe_3_O_4_ nanoparticles and amine functionalized CNCs, which did not produce a stable colloidal solution due to the lack of thiol group in this system. Thus for CNC with no thiol group, we observed two population of nanocages (Fig. 4d), however for CNC with thiol group, we found single population of nanocages (Fig. 4c) indicating the importance of thiol conjugation to CNC-amines. From the high magnification micrograph by HRTEM as shown in Supplementary Figure 2b, the crystal planes of these CNC-encaged Fe_3_O_4_ nanoparticles were clearly visible. The magnetite phase of Fe_3_O_4_ nanoparticles can be identified from the d spacing = 2.9 Å, which is due to (220) crystal orientation that clearly proved presence of Fe_3_O_4_ within the nanocage cluster (Supplementary Figure 2b)^41^. Magnetic properties measurement was conducted to validate the magnetic nature of iron oxide nanoparticle bound CNC nanocages (Supplementary Figure 6), and found that, an ordinary magnet with its nominal field strength (as low as ~35 Gauss) can easily separate out the synthesized magnetic nanocages demonstrating its magnetic nature of very high magnetic moment. In following steps, we immobilized Tf onto CNC surface by first functionalizing Tf with succinic anhydride, and then connecting succinylated Tf onto CNC-NH_2_ using carbodiimide chemistry. The successful immobilization of Tf onto CNC surface was validated by Bradford assay (Supplementary Figure 4). We adopted Tf conjugation as the final step to ensure that Tf structures are not adversely affected by iminothiolane modification necessary for connecting Fe_3_O_4_ NPs to CNCs. Overall, this set of DLS and zeta potential measurement revealed that amination and consequent thiolation of CNC is necessary to form stable colloidal suspension of Fe_3_O_4_-CNC magnetic system amenable to post-synthetic modification with Tf for CTC capture.

### Cell-capture efficiency of CNC nanocages using artificial CTC suspension

To evaluate the efficiency of CNC nanocages and to account the capture efficiency, an artificial cell suspension was prepared first by spiking 25, 50, and 100 Tf-positive HCT116 cells in PBS and treated with CNC nanocage samples (1A-1C). Among these samples, 1 A and 1B contained nearly equivalent content of Tf, but varying amount of Fe_3_O_4_ nanoparticles, while sample 1 C contained reduced amount of Tf at higher Fe_3_O_4_ loading content than both 1 A or 1B formulations (Tab le 1 and Supplementary Table-1). We also included two controls, i.e. CNC-Fe_3_O_4_^(−SH, +Tf)^ (sample 1D), for which Fe_3_O_4_ is stabilized onto the nanocages without metal-thiol bond, thus forming an unstable colloidal dispersion and a no-Tf control, CNC-Fe_3_O_4_^(+SH, −Tf)^. For cell capture efficiency, we optimized Tf content in CNC nanocages from 0.038 mg to 0.028 mg Tf/mg of CNC for samples 1 A to 1 C. The captured cells were stained with DAPI and eosin and the images were captured using fluorescence microscope. After paramagnetic isolation, both captured and uncaptured cells were accounted for each sample (Table 1). The capturing efficiency was measured from the ratio of captured and uncaptured cells following a reported methodology^83^. CNC nanocages with Tf moieties were expected to achieve enhanced interaction with Tf-positive HCT 116 cells. Table 1 shows Tf content in CNCs versus % cell capturing efficiency using artificial cell suspension. As observed from the table, between sample 1B and sample 1 C, sample with higher Tf content demonstrated higher cell capturing efficiency. Cell capture efficiency reduced significantly for CNC-Fe_3_O_4_^(−SH, +Tf)^ (1D), where the Fe_3_O_4_ nanoparticles are physically attached inside CNC nanocages without metal-thiol stabilization. Almost nominal numbers of CTCs were captured for no-Tf control samples, i.e. CNC-Fe_3_O_4_^(+SH, −Tf)^ systems. To understand the role of Fe_3_O_4_ content, we compared sample 1 A and 1B, which contain 2 mg and 2.5 mg of Fe_3_O_4_/mg CNC respectively. As the metal content in 1B is 20%, higher than that of in 1 A, under same incubation time of 5 minutes under magnetic induction, 1B showed enhanced efficiency of CTC cell capture, which is well separated. Therefore, despite containing nearly equivalent content of Tf, significant increase in the capture efficiency was noticed for sample 1B.

**Table 1:** Composition of tested samples and cells capturing efficiency of CNC nanocages using HCT 116.

Sample	Tf content (mg Tf mg^−1^ CNC)	Fe -content (mg Fe mg^−1^ CNC)	Cells added			
			25	50	100	
1A	0.038	2.0	15 (60%)	32 (64%)	65 (65%)	Cells captured
1B	0.036	2.5	21 (84%)	36 (72%)	76 (76%)	
1C	0.028	3.0	13 (52%)	29 (58%)	56 (56%)	
1D CNC-Fe_3_O_4_^(−SH, +Tf)^	0.017	1.3	2 (8%)	2 (4%)	5 (5%)	
CNC-Fe_3_O_4_^(+SH, -Tf)^	0.00	1.4	1 (4%)	3 (6%)	7 (7%)	

### Enumeration of CTC capturing efficiency for Tf-CNCs with HNC patient blood samples

It is very challenging to detect, isolate and enumerate CTCs from whole blood from patient sample. The process is inherently complicated because of low occurrence of CTCs in the background of a large number of non-target cells such as erythrocytes and leukocytes in blood^84^. The challenges also includes lack of sensitive and specific biomarkers that can be used for isolation of CTCs. Circulating tumor cells can be positively isolated based on EpCAM overexpression, however minimizing non-specific cell-capture remains a prudent challenge. The current-CTC enriching technologies include use of specific antibodies that bind selective surface markers on CTCs^69^. As most tumors originate from epithelial cells, hence epithelial cell markers can be used to isolate CTCs from multitude of other blood cells. For example, EpCAM, cytokeratin (CK) 8, 18, 19, and transferrin (Tf) are epithelial cell surface markers, which are overexpressed in cancer cells of epithelial origin^84,85^.

**Figure 5:**
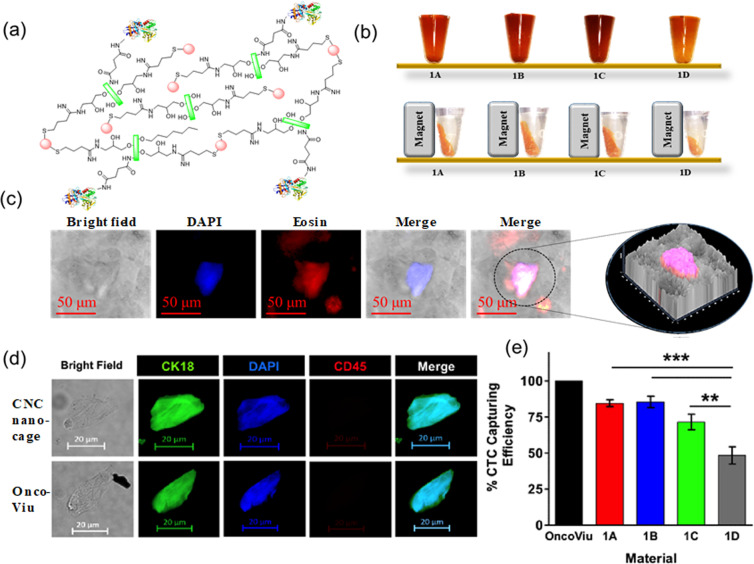
(**a**) Schematic representation of magnetic nanocages where CNC-NH_2_ is used as the major component of a matrix network immobilized with iron oxide nanoparticles and Tf. (**b**) Sample 1 A,1B,1 C and 1D (CNC-Fe_3_O_4_^(−SH, +Tf)^) were in contact with a strong magnet showing the accumulation of nanocages near the magnetic field. (**c**) Representative image of HCT 116 captured using CNC nanocages, stained with DAPI and eosin. Few peaks were observed adjacent to captured cell in 2.5D image. (**d**) Representative fluorescence images of circulating tumor cells isolated by Oncoviu and CNC nanocages from blood sample of HNC cancer patients (under 63X objective). Antibodies recognizing CK18 and CD45 are in green and red, respectively. Nuclei are stained with DAPI (blue), and multichannel composite merged. (**e**) CTC capturing efficiency (%) of CNC-1A -1B -1C -1D relative to Oncoviu (n = 6, 1-way ANOVA, Bonferroni Multiple Comparison post-hoc test *p < 0.05, **p < 0.01, or ***p < 0.001 mean ± SEM).

Figure 5(a–e) illustrates the materials, method and isolation result of CTCs from de-identified blood samples of patients with HNC. Captured cells were isolated and enumerated under the influence of magnetic field. While new CTC enumeration advanced materials are being explored, they remain to be compared with clinically developed platforms. Hence we compared our Fe_3_O_4_-CNC nanocages with OncoDiscover CTC Liquid Biopsy Technology (Actorius Innovations and Research), which uses Oncoviu kit for detection, capture and enumeration of CTC from peripheral blood^69,86^. Oncoviu uses EpCAM antibody-coated magnetic ferrofluid to enrich CTCs from peripheral blood and has been clinically validated with regulatory approvals. For cells captured with CNC nanocages, immunostaining was performed with anti-CK18, anti-CD45, and DAPI to identify CTCs (CK18^+^ CD45^-^ DAPI^+^) and distinguish the cell population from leukocytes (CK18^−^ CD45^+^ DAPI^+^) (Fig. 5d). Furthermore, no CTC was observed in healthy individual’s blood sample, highlighting the selectivity and specificity of CNC-derived material. We observed that CTC capturing efficiency varied across different CNC nanocage samples (1A-1C) and the controls [i.e. CNC-Fe_3_O_4_^(−SH, +Tf)^ and CNC-Fe_3_O_4_^(+SH, −Tf)^], depending on the loading content and stabilization mode of Tf and Fe_3_O_4_ inside the nanocage. For instance, nanocage sample 1A was found to have a capture efficiency of 84.47 ± 2.361%, while for samples 1B and 1C, CTC capturing efficiency was found to be 85.41 ± 3.993% and 71.5 ± 5.37%, respectively. The cell capturing of these samples was significantly higher compared to control CNC-Fe_3_O_4_^(−SH, +Tf)^ (48.37 ± 5.979%) (Fig. 5e). While comparing Table 1 and Fig. 5e, it is evident that CTC capturing efficiency was varied from sample 1A-1C, clearly indicating that the capturing efficiency is dually directed by the surface presentation of Tf and by Fe_3_O_4_ nanoparticle content within the nanocage network for patient blood samples as well. For both HCT 116 cells and in HNC patient blood, samples 1A and 1B, containing 0.038 and 0.036 mg Tf mg^−1^ of CNCs respectively, showed a higher capture efficiency of CTCs compared to samples 1C bearing 0.028 mg Tf mg^−1^ of CNCs. Despite increasing the content of iron oxide nanoparticles, CTC capturing efficiency decreased from sample 1B to sample 1C due to a relatively low Tf content. In Tf-bearing control sample where Fe_3_O_4_ is not stabilized by metal-thiol bond (i.e. CNC-Fe_3_O_4_^(−SH, +Tf)^ system, 1D), showed lower capture efficiency for CTCs. This observation indicates that the presence of thiol group helps to stabilize Fe_3_O_4_ nanoparticles within the CNC structures also contributes to magnetic capture of CTCs. Thus, the experimental data suggests that both Tf and Fe_3_O_4_ content in CNC nanocage plays decisive roles in CTC capture efficiency through magnetic induction. From chemical standpoint, incorporation of amine functionalization, and subsequent conversion of the resulting amine groups to thiol is the key driver for governing Fe_3_O_4_ and Tf amount that can be conjugated to the system. IR spectroscopy (Supplementary Figure 7) indicates the linear dependence of Fe_3_O_4_ immobilization with the amount of −SH groups present within the CNC nanocage. From eosin and DAPI staining presented in Fig. 5, it was evident that captured CTCs were viable on CNC scaffold as observed by the presence of intact nucleus. Cytotoxicity of iron oxide conjugated CNC nanocages were also validated against mammalian fibroblast cell lines (HFF2) using MTT assay (Supplementary Figure 8). These data shows that CNC nanocages bearing iron oxide nanoparticle are compatible both with circulating cancer cells and healthy cells.

## Discussions

We designed and evaluated the efficiency of a CNC-based diagnostic platform towards enumeration of CTCs. The CTC detection technology has been established with multiple utilities in cancer therapy with diagnostic, prognostic value, and for monitoring of early metastasis and relapse of the disease. Especially, HNC is of interest as the cancer of the upper aero-digestive tract is the sixth most common cancer globally and is estimated to account for 4.8% of all cancers. Conversely, the squamous cell carcinoma of the head and neck (HNSCC) accounts more than 95% of these tumours. In spite of surgical resection with negative histopathological margins, around 20% of HNC patients demonstrate locoregional recurrence. In oral cavity cancer, about 23–27% of HNSCC patients with clinical follow-up are indicative of micro-metastatic development, and the presence of lymphatic metastases is thus a reliable indicator of a poor prognosis and its occurrence decreases the survival of patients by 50%^87^. With these statistical facts in mind and considering the potential risk of locoregional or distant recurrence, we envisioned to develop a low-cost CTC detection platform that will have beneficial implications for HNC patients.

We report for the first time, an orthogonal chemical modification strategy of CNCs to generate a cross-linked functional network stabilized by Fe_3_O_4_ nanoparticles resulting in the formation of a magnetic ‘nanocage’. These network structures can be further modified with Tf for developing point-of-care diagnostic platform for CTCs. It has been an well-established fact that multiple hydroxyl groups of CNCs provided superior water dispersibility of the system, and presents multivalent anchor points for Tf protein along the CNC surface. In our hand, these features of CNCs enabled stable and viable interactions between the nanocage and CTCs. Cell isolation and enumeration capacity of these CNC-derived constructs were found to be dependent on the density of the cell recognizing Tf ligand immobilized onto the nanocages, as well as on the concentration of Fe_3_O_4_ NPs that served as network stabilizer as well as the driver for magnetic separation of cells. *In-situ* magnetic separation and the efficiency of nanocages to capture CTCs were validated for HCT 116 cell lines. Blood samples of clinical HNC patients were analyzed and CTCs were precisely enumerated and compared against a clinically relevant standard, such as Oncoviu kit, which uses EpCAM as surface marker CTC capture motif. Thus, it is obvious that Oncoviu showed greater specificity than our CNC-Fe_3_O_4_ systems (Fig. 5e). However, use of Tf in CNC-Fe_3_O_4_ system can provide a significant advantage over antibody-mediated capture in terms of de-complexation of CTCs from the scaffold for further analysis. As organoid culture is becoming a more relevant tool for omics-based analysis of genetic, RNA and protein status of cells in their native milieu, mild detachment of cells from the capturing scaffold will become more critical to rescue cellular phenotypes. CTC-Tf receptor interactions could be easily de-complexed unlike the antigen-antibody mediated interactions that will take place between CTCs and EpCAM-engineered systems. This will be of greater importance as the de-coupled CTCs from CNC scaffold would show better proliferation potential without any cell surface implications. Thus, Tf like proteins may offer better choice in further characterization of CTCs than antibody-driven capture platforms. Currently, we are exploring the mechanism through which of CTCs interact with the CNC surface. We envision that use of cellulose-derived materials can be employed as a low-cost, commercially viable option to fabricate ‘point-of-care’ diagnostics for cancer prognosis and monitoring.

## Methods

### Materials

Cellulose nanocrystals (CNC) were obtained from University of Maine, ME, USA (CAS 9004-34-6), Ferric chloride hexahydrate (CAS 10025-77-1), Ferrous chloride tetrahydrate (CAS 13478-10-9), Fluorescein isothiocyanate (CAS 27072-45-3), Traut’s reagent (CAS 4781-83-3) and Transferrin (CAS 11096-37-0) were procured from Sigma Aldrich, USA. All reagents and solvents were from Millipore Sigma unless otherwise specified. All reagents were used as received unless otherwise stated.

### Synthesis of Fe_3_O_4_ immobilized magnetic nanocages

Magnetic nanocages were realized via a two-step synthetic route. First, CNCs were functionalized with primary amine groups, and in the second stage, Fe_3_O_4_ NPs, and finally Tf was immobilized onto crystal surfaces as described below:

### Amine and Thiol group introduction on CNC surface

For functionalization of CNCs, previously reported procedure described by Dong *et al*. has been adopted^29^. The crystals were treated with varying concentration of epichlorohydrin (12, 24 and 48 mmol/g cellulose) at 60 °C for 2 h in alkaline conditions. The reaction mixture was then treated with ammonium hydroxide and reacted for additional 2 h at 60 °C. Dialysis of the mixture was performed until the pH of the dialysate containing amine functionalized CNC (CNC-NH_2_) reaches 7.0. Successful generation of amine groups onto CNC surface was validated by UV-Vis spectroscopy, N/C ratio determination through elemental analysis, FTIR and XPS spectroscopy. Addition of thiol groups to generate CNC-SH from CNC-NH_2_ was achieved through Trout’s reaction. In this process, 0.1 L of PBS solution and 2.5 mL of EDTA were mixed vigorously and adjusted to pH 7.6 by adding concentrated NaOH. This coupling buffer (950 μL) was then added to 4 mL of CNC-NH_2_ (2 mg/mL) solution. Traut’s reagent (2-iminothiolane) (2 mg) was added to 1 mL of the coupling buffer and was transferred 50uL to CNC-NH_2_ solution. The reaction medium was incubated for 45 min at room temperature after which it was centrifuged at 2000 rpm for 5 min and washed with water. Thiol functionalized CNC pellet was collected for further modification with Fe_3_O_4_ NPs and Tf.

### Immobilization of Fe_3_O_4_ NPs and Tf on amine functionalized CNCs

Synthesis and characterization of NPs for immobilization onto CNCs were conducted following a previously reported procedure via hydrothermal method^75^. Freshly prepared, 400 μL of Fe_3_O_4_ NPs in DI water (5.3 mg/mL) was added dropwise to CNC-SH suspension under bath sonication over 20 minutes. Conjugation of Tf onto the residual amines of CNC-SH was realized via a two-step process. In the first step, Tf was succinylated in alkaline conditions to generate Tf-succinate which was then coupled to the amine groups on CNC surface through carbodiimide mediated amide forming reaction. Buffer solution 1 M NaHCO_3_ of 134 μL added to 2.0 mg of Tf at room temperature and mixed by vortex mixture. Succinic anhydride (3.0 mg) was added to the mixture and kept at room temperature for 30 min after maintaining pH of 8 by addition of 0.1 M NaOH. Distilled water of 2 mL was added to this mixture and was transferred to the suspension containing Fe_3_O_4_ NPs immobilized CNCs. In the final step, 2.0 mg of EDC and 2.0 mg of NHS were added to the final sample. Four different types of modified CNC samples (1A-1D, shown in Table 1 and Supplementary Table-1) were prepared and used as CTC capturing scaffolds. The content of Fe_3_O_4_ NPs were gradually increased from sample 1A-1C by keeping CNC and Tf content constant. Thiolation was not conducted on sample 1D, so as to evaluate and compare the CTC capturing performance of non-covalently encapsulated Fe_3_O_4_ NPs within the CNC nanocages.

### Cell culture

HCT116 cells of passage number 40 were procured from (NCCS, Pune, India) and cultured in McCoy media (HiMedia), supplemented with 10% fetal bovine serum (Invitrogen) and 1% antibiotic (Penicillin 100 μg/mL, streptomycin 100 μg/mL, Sigma).

### Magnetic property analysis of CNC samples

Samples were 3X diluted with Milli-Q water and kept in contact with magnet for 15 min. Images were captured using Dino Light camera.

### Estimation of capture efficiency from artificial circulating tumor cells (CTC) suspension

Artificial cell suspension was prepared by spiking 25, 50 and 100 human colon cancer cell line (HCT116) cells in PBS. HCT 116 cell suspension was treated with CNC-nanocage samples, 1 A (2.1 μL/mL), 1B (3.15 μL/mL), 1 C (2.21 μL/mL), and 1D (2.2 μL/mL) for 1 h. After 2 min incubation of cells with nanocages, the samples were subjected to magnetic field separation. A cell pellet was obtained from individual samples under the strong magnetic field. The pellet was washed with PBS three times, and after removal of magnetic field, the suspension was added into 96 well plate. Captured cells were stained with DAPI and eosin and the cells were counted using fluorescence microscope (Zeiss Axio Observer A1). Finally, the cell capturing efficiency was calculated for individual samples.

### CTC capture efficiency in HNC patient blood samples

Blood samples of 5 mL were drawn from deidentified patients with head and neck cancer at TATA Memorial Hospital (TMH), Mumbai, India. Institutional Ethical Committee of TATA Memorial Hospital (IEC/1117/1903/001) approved clinical blood-sampling procedures and informed consent were obtained for patient participation. These protocols were approved by Institutional Ethical Committee of TATA Memorial Hospital, and the methods were carried out in accordance with the relevant guidelines and regulations. Blood sample from the head and neck cancer patients as well as that from healthy individuals were subjected to red blood cell lysis for 10 min and centrifuged at 2000 rpm for 15 min. The cell pellet was resuspended in 1X PBS and incubated with Oncoviu or CNC nanocage samples 1 A, 1B, 1 C or controls. The cell suspension was exposed to magnetic field in order to isolate captured CTC fractions. The captured CTC fraction from individual samples was immuno-stained with anti-CK18 (Cytokeratin 18), anti-CD45 (leukocyte common antigen) and DAPI (4’, 6-diamidino-2-phenylindole; nucleus stain). Captured CTCs were identified and enumerated by fluorescence microscopy imaging (ZEISS, Axio Observer Z1), and images were processed with ZEISS Pro software from ZEISS.

## Supplementary information

Supplementary Information.

## Data Availability

All relevant data are included in this Article and its Supplementary Information files.
