# Author Correction: Cellulose Mediated Transferrin Nanocages for Enumeration of Circulating Tumor Cells for Head and Neck Cancer

**DOI:** 10.1038/s41598-020-72920-9

**Published:** 2020-10-01

**Authors:** Raj Shankar Hazra, Narendra Kale, Gourishankar Aland, Burhanuddin Qayyumi, Dipankar Mitra, Long Jiang, Dilpreet Bajwa, Jayant Khandare, Pankaj Chaturvedi, Mohiuddin Quadir

**Affiliations:** 1 Department of Mechanical Engineering, Materials and Nanotechnology Program, North Dakota State University, Fargo, ND 58108 USA; 2 Department of Coatings and Polymeric Materials, North Dakota State University, Fargo, ND 58108 USA; 3 School of Pharmacy, Maharashtra Institute of Technology-WPU, Pune, India; 4 Actorius Innovations and Research (AIR) Pvt. Ltd, Pune, India; 5 Department of Electrical and Computer Engineering, North Dakota State University, Fargo, ND 58108 USA; 6 Department of Medical Oncology, Tata Memorial Hospital, Mumbai, 400012 Maharashtra India; 7 Department of Mechanical and Industrial Engineering, Montana State University, Bozeman, MT 59717-3800 USA

Correction to: *Scientific Reports* https://doi.org/10.1038/s41598-020-66625-2, published online on 19 June 2020

In the original version of this Article, Dilpreet Bajwa was incorrectly affiliated with ‘Department of Mechanical Engineering, Materials and Nanotechnology Program, North Dakota State University, Fargo, 58108, ND, USA’. The correct affiliation is listed below.

Department of Mechanical and Industrial Engineering, Montana State University, Bozeman, MT, 59717–3800, USA.

This error has now been corrected in the PDF and HTML versions of the Article and in the accompanying Supplementary Information file.

