# Supplementary material for: Cellulose Mediated Transferrin Nanocages for Enumeration of Circulating Tumor Cells for Head and Neck Cancer
Source: Sci Rep. 2020 Jun 19;10:10010. doi: 10.1038/s41598-020-66625-2 (PMC7305211; doi:10.1038/s41598-020-66625-2)
Supplement: Supplementary file 1 — Supplementary Information. [file 41598_2020_66625_MOESM1_ESM.pdf]

Supplementary Information

Cellulose Mediated Transferrin Nanocages for Enumeration of Circulating Tumor Cells for Head and Neck Cancer

Raj Shankar Hazra [1], Narendra Kale [3], Gourishankar Aland [4], Burhanuddin Qayyumi [6], Dipankar Mitra [5], Long Jiang [1], Dilpreet Bajwa [1], Jayant Khandare [3, 4], Pankaj Chaturvedi [6], Mohiuddin Quadir [2]**

[1] Department of Mechanical Engineering, Materials and Nanotechnology Program, North Dakota State University, Fargo 58108, ND, USA

[2] Department of Coatings and Polymeric Materials, North Dakota State University, Fargo 58108, ND, USA

[3] Maharashtra Institute of Technology-WPU, School of Pharmacy, Pune, India

[4] Actorius Innovations and Research (AIR) Pvt. Ltd., Pune, India

[5] Department of Electrical and Computer Engineering, North Dakota State University, Fargo 58108, ND, USA

[6] Department of Medical Oncology, Tata Memorial Hospital, Mumbai-400012, Maharashtra, India.

Characterization of CNC functionalized with amine

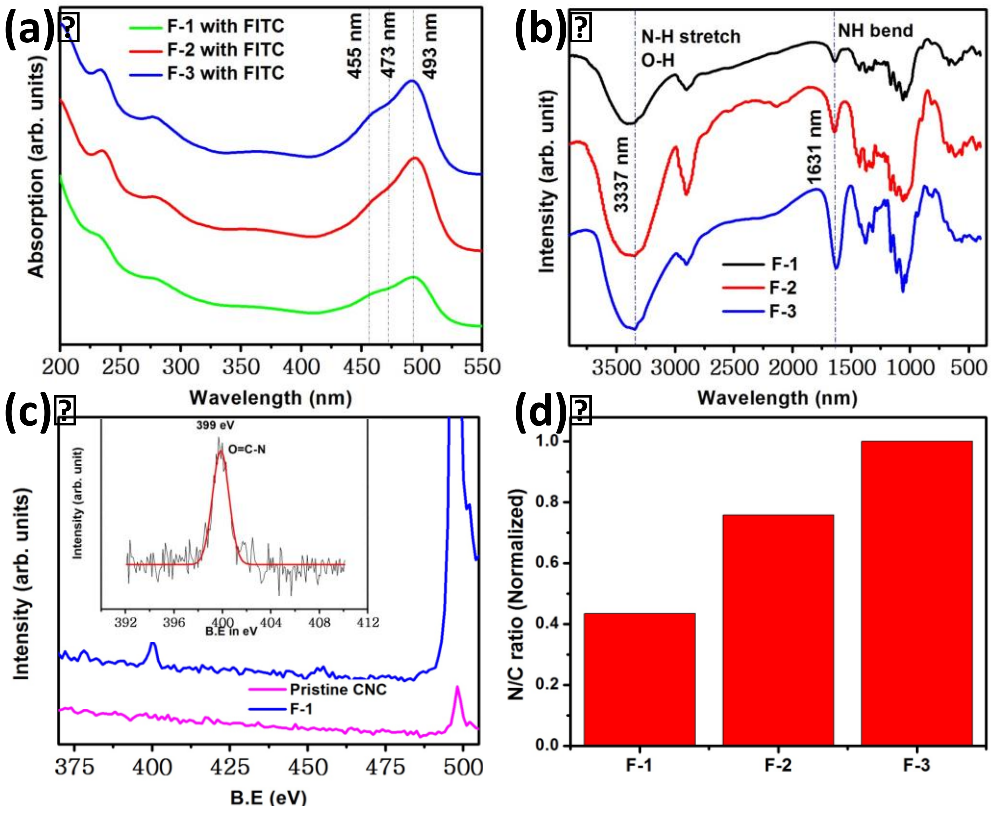

Supplementary Figure 1: Characterizations of modified CNC samples to monitor nitrogen doping (CNC-NH_2 functionalization). (a) UV-Vis spectra of three modified CNC samples (F-1, F-2 and F-3) with FITC labelling prepared along the formulation provided. (b) IR spectra of modified CNC samples (F-1, F-2 and F-3) showing N-H stretching and bending peaks. (c) XPS spectroscopy on pristine CNC and amine based modified CNC (high magnification scans on amine based modified CNC is shown in the inset figure). (d) Elemental analysis by combustion using automatic analyzer to measure N/C ratio of F-1, F-2 and F-3 CNC samples indicating gradually increasing numbers of amine groups present across the samples.

Characterization of iron oxide nanoparticles: DLS and HRTEM analysis.

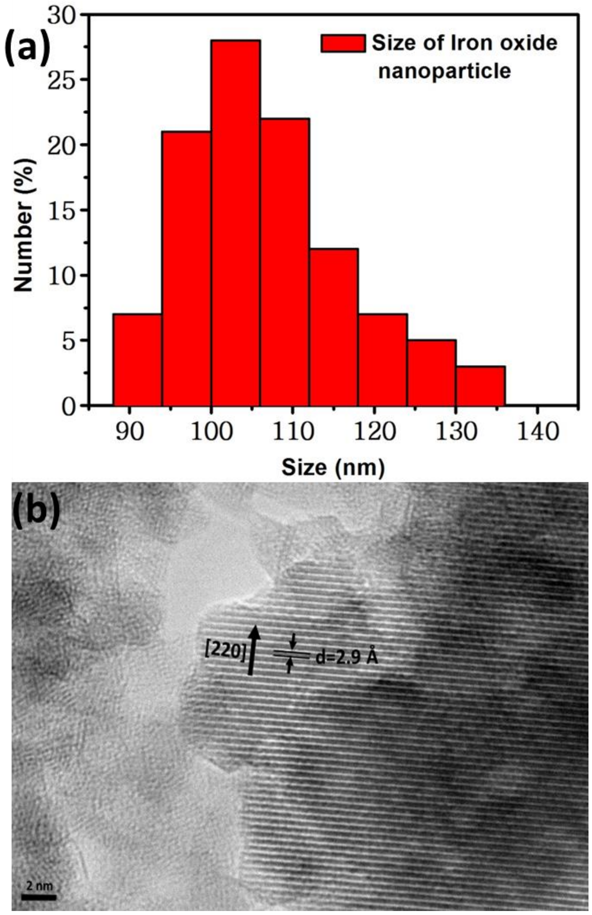

Supplementary Figure 2: (a) Particle size distribution of Fe_3O_4 nanoparticles as observed by DLS. (b) High magnification micrograph of HRTEM where the crystal planes of the nanoparticles are clearly visible, indicating the magnetite phase of Fe_3O_4 identifiable from the d-spacing of 2.9 Å.

Quantification of iron oxide loading content in nanocage

Measurement of iron oxide nanoparticle content has done using calibration curve analysed from UV-Vis spectroscopy at 370nm absorption. This calibration curve is used to quantify the iron oxide loading in nanocage under water suspension condition.

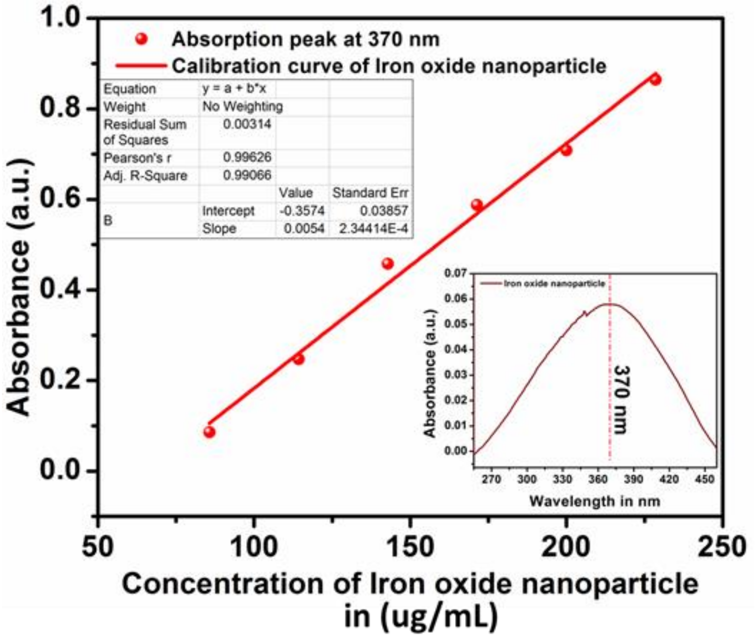

Supplementary Figure 3: Calibration curve for quantification of iron oxide nanoparticle.

Quantification of transferrin (Tf) loading content in nanocage

Bradford assay calibration curve is done with measuring absorbance at 595nm of standard protein BSA 2mg/ml and series of diluted concentration with Bradford dye reagent. Tf protein loading concentration in nanocage is measured from the Bradford calibration curve.

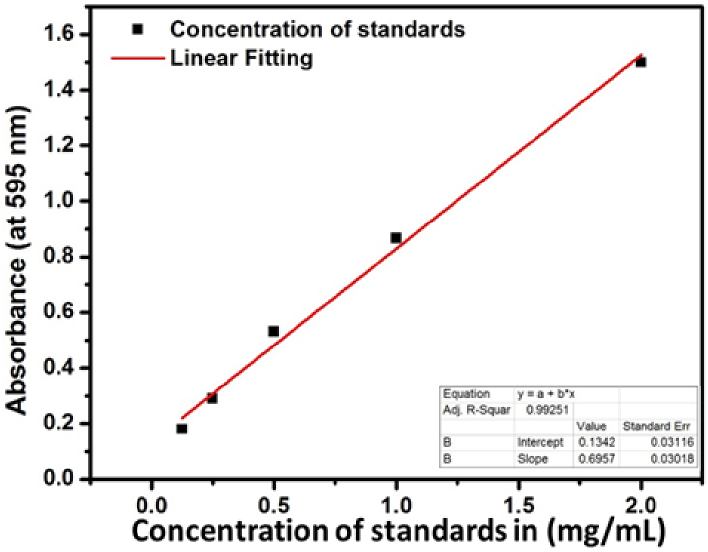

Supplementary Figure 4: Calibration curve for modified Bradford assay.

Composition of Tf-functionalized CNC nanocages encapsulating iron oxide nanoparticles

CNCs were treated with 96 mmol/g cellulose of epichlorohydrin at 60°C for 2h in alkaline conditions. The reaction mixture was then treated with ammonium hydroxide and reacted for additional 2h at 60°C. Dialysis of the mixture was performed until the pH of the dialysate containing amine functionalized CNC (CNC-NH_2) reaches 7.0. All samples shown in table below have same level of functionalization with primary amine. Traut's reagent was added to CNC-NH_2 in the presence of 1.9mL EDTA/PBS buffer solution at varying concentration.

Supplementary Table-1. Composition of Tf-functionalized CNC nanocages encapsulating iron oxide nanoparticles

Sample	CNC content (mg)	Traut's reagent with EDTA/PBS buffer (uL) used	Iron oxide in CNC (mg Fe mg^{-1} CNC) immobilized	Transferrin in CNC (mg Tf mg^{-1} CNC) immobilized
1A CNC-$Fe_3O_4^{(+SH, +Tf)}$	16	50	2.0	0.038
1B CNC-$Fe_3O_4^{(+SH, +Tf)}$	16	75	2.5	0.036
1C CNC-$Fe_3O_4^{(+SH, +Tf)}$	16	100	3.0	0.028
1D CNC-$Fe_3O_4^{(-SH, +Tf)}$	35	0	1.3	0.017
CNC-$Fe_3O_4^{(+SH, -Tf)}$	35	100	1.4	0.000

TEM images of iron oxide conjugated CNC nanocage

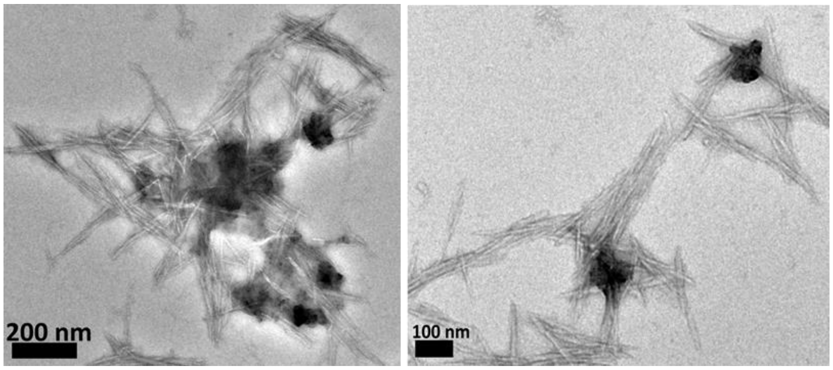

Supplementary Figure 5: Additional TEM images of Fe_3O_4 conjugated nanocage which shows iron oxide conjugated to thiolated CNCs to form magnetic nanocage

Magnetic measurement of Iron oxide conjugated nanocage

We have demonstrated a calibration of the magnetic field strength induced on the iron oxide nanoparticle by using a Gauss/Tesla meter (Company name 5170), where the distance between the magnet and the sample is varied from 1 to 4 cm (Supplementary Figure 6-A). The fitting data shows that magnetic field strength is proportional to Inverse Square of distance, which is observed due to magnetic nature of the stable colloidal materials of Fe_3O_4-CNC nanocage systems (Supplementary Figure 6-B). This result clearly shows that an ordinary magnet with its nominal field strength (as low as ~ 35 Gauss) can easily separate out the synthesized magnetic nanoparticle demonstrating its magnetic nature of very high magnetic moment. We have shown 6000 Gauss magnet (Accu-Band 6000 Rec Magnets, Gold Plated) separated iron oxide conjugated nanocage from the suspension (Supplementary Figure 6-C). This experiment indicates that magnetic property of Fe_3O_4 NP is not diminished due to CNC entrapment. This observation is in accordance with previously published literature which shows the magnetic properties of free Fe_3O_4 or polymer modified Fe_3O_4 species.

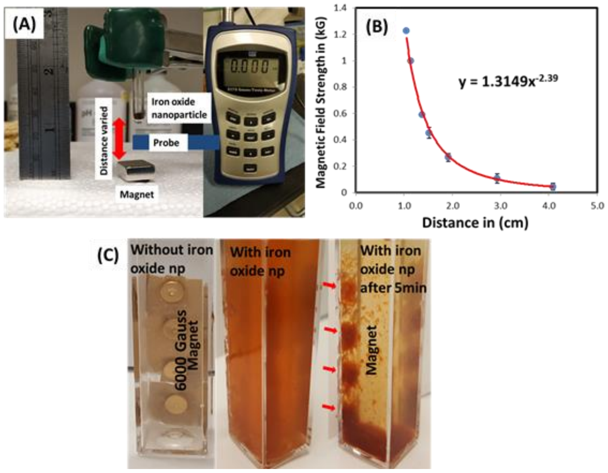

Supplementary Figure 6: (A) Magnetic field strength measurement with varying distance between the magnet and the sample (B) Magnetic field strength and distance dependence (C) 6000 Gauss magnet separated iron oxide conjugated nanocage from suspension.

Iron oxide loading dependence of degree of thiol group functionalization level

FTIR analysis on iron oxide conjugated cellulose nanocage is performed where thiol modification increased from F1 to F3 (F3>F2>F1) (Keeping iron oxide content constant for each formulation). Bending at 2600 cm^{-1} comes due to SH stretching vibration. The low bending vibrational intensity for F1 represents iron oxide is binding with S group and diminish SH stretching vibration. In case of F2 and F3, there are higher SH stretching vibration which comes due to presence of remaining thiol group after conjugating certain content of iron oxide nanoparticle. F2 and F3 can still load more iron oxide nanoparticle before completely diminish SH stretching vibration.

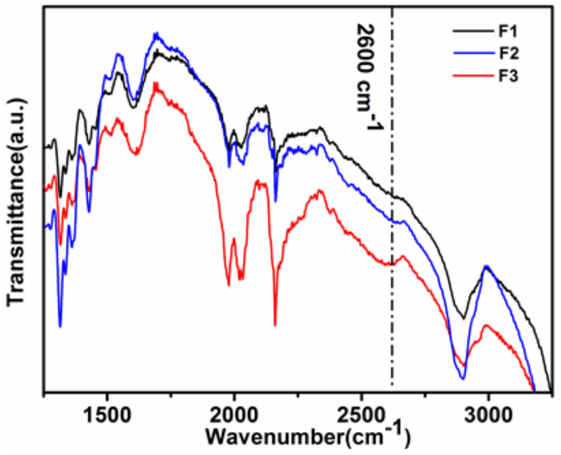

Supplementary Figure 7: FTIR analysis on iron oxide conjugated cellulose nanocage where thiol modification increased from F1 to F3 (F3>F2>F1) while maintaining iron oxide content constant for each formulation).

Cell viability study of magnetic nanocage

Cell viability study has done by growing human foreskin fibroblasts (HFF 2) with 10,000 cells/well in 96 well plate with DMEM containing FBS (10%) and Penicillin/Streptomycin (1%) for 24 hr at 37°C, 5% CO_2. Treatment of iron oxide loaded cellulose nanocage sample has done with treating series of diluted concentration (7.48, 6.32, 5.53, 4.74, 3.95, 3.16, 2.37, 1.58 and 0.79 ug/mL) for another 24hr at 37°C, 5% CO_2. Cell viability was measured with Alamar blue assay by treating 20ul to 200ul of well volume for 4hr of incubation (at 37°C, 5% CO_2). Measurement of the fluorescence has done at 545nm excitation and 590 emission wavelength.

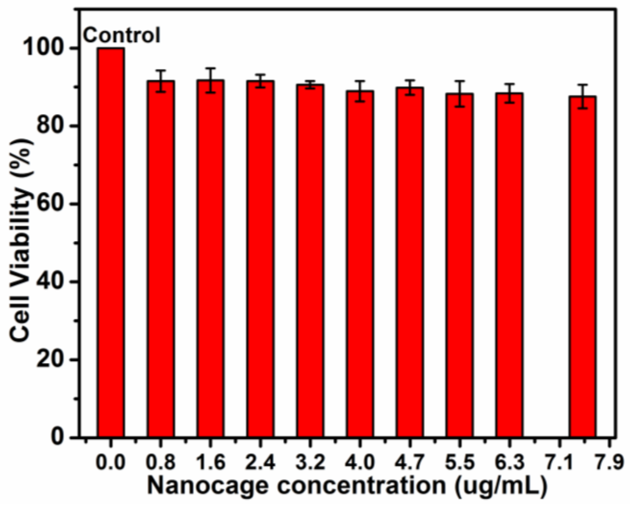

Supplementary Figure 8: Cell viability study of iron oxide conjugated cellulose nanocage in healthy mammalian fibroblast cell lines (HFF2) using MTT assay.
